# Recognition in interaction: theoretical and empirical observations

**DOI:** 10.3389/fsoc.2023.1223203

**Published:** 2024-01-23

**Authors:** Emmi Koskinen, Arto Laitinen, Melisa Stevanovic

**Affiliations:** ^1^Faculty of Social Sciences, University of Helsinki, Helsinki, Finland; ^2^Faculty of Social Sciences, Tampere University, Tampere, Finland

**Keywords:** conversation analysis, recognition, misrecognition, interaction, sociological theory, solidarity, affiliation

## Abstract

In the current paper we aim to combine the theoretical ideas of recognition theory to conversation analytical, empirical observations. We ask what recognition theories can give to conversation analysis, and vice versa. We operate on a model of recognition that consists of three different modes: respect, esteem, and love/care, and which distinguishes the levels of conversational actions and the attitudes of recognition manifested in such actions. In this study we examine data examples from various conversational settings (institutional, quasi-experimental, family interaction) and activities (decision-making, storytelling), focusing on the more complex cases of (mis)recognition. We show how recognition can appear both explicitly and implicitly in conversational sequences, and demonstrate how the levels of conversational actions and recognition can be either congruent or incongruent with each other. At the end of the article, we discuss the implications of this view for the interface of conversation analysis and sociological theory, arguing that it can inform and promote the development of interactionally based social and societal critique.

## 1 Introduction

The philosophy of recognition (*Anerkennung*) originates with Fichte and Hegel, who have theorized the social conditions of becoming a person. They claim that to be a self-conscious, free agent, one must be recognized by other self-conscious, free agents. In recognizing someone, a person limits their own activities accordingly: lets the other be free. In contemporary social and sociological theory, a recognition-theoretical approach to human existence has been advanced by, for example, Charles Taylor and Axel Honneth. Taylor (1992, p. 26) called recognition a “vital human need” and made a distinction between two forms of recognition: *difference-blind* politics of universality and *difference-sensitive* politics of difference (Taylor, 1995; see Laitinen, 2002). In Honneth’s theory (1995) three forms of recognition and self-relation were thematized: (1) respect and self-respect (2) social esteem and self esteem, and (3) love and self-confidence. Honneth chose these three possible modes based on their explanatory and normative relevance in relation to critical social theory (Laitinen, 2002, p. 470). He paid special attention to the normative expectations of recognition, and the experiences of suffering from misrecognition, insults, and subordination.

In interaction theory, the idea of recognizing each other’s selves, while simultaneously letting others be free from imposition, resonates with Brown and Levinson’s (1987) concepts of *negative* and *positive politeness.* Brown and Levinson have argued that interactants use various politeness strategies to protect each other’s social self-images, which they named as positive and negative *face* (cf. Goffman, 1955). Negative face refers to the human desire to be free from imposition, whereas positive face refers to the human desire to be validated by others. In recognition theoretical terms, negative politeness means freedom from misrecognition/mistreatment, and positive politeness the presence of positive recognition; positive affirmation of one’s dignity (respect), merits (esteem), special standing (love/care). Brown and Levinson’s framework, however, only operates at the level of the design of individual actions or turns of talk (e.g., linguistic form). Conversation Analysis (CA) has brought the empirical analysis of these human desires to the level of *sequences* of talk (Clayman, 2002, p. 230). Politeness theory has been of relevance, for example, in the sequential analysis of preference structure and the maintenance of *social solidarity* and *affiliation* in and through interaction (Clayman, 2002).

CA is a qualitative methodological program for studying video recordings of interactions with an aim to unravel the reoccurring interactional practices through which social actions are constructed (e.g., Schegloff, 2007; Sidnell and Stivers, 2013). The structural analysis of action in ordinary conversation relies on the notion that social interaction is informed by institutionalized structural organizations of practices to which participants are normatively oriented (Heritage, 2008, p. 303). It is this structural assumption, which is fundamentally associated with Goffman’s (1974) ‘Interaction Order’, that differentiates CA as an approach to the study of social action from, for example, sociolinguistics or the sociology of language (Heritage, 2008, p. 303). CA offers a ‘procedural approach’ to social action, operating on the level of sequential organization (Clayman, 2002, p. 230). CA is thus its own enterprise, which focuses on talk-in-interaction embedded in sequential context, and the orderliness that participants produce and to which they demonstrate their orientations (Maynard, 2013, p. 28). Initially CA was a radically empirical enterprise (Haakana et al., 2009). A founding principle has been not to impose external ideas or theories on the data but to focus on how the participants themselves orient to interactional phenomena. In our current endeavor, however, we follow the authors such as Linell (2009) and Svennevig (2014), who have argued that many analytically interesting questions “go beyond the members’ perspective and call for situation-transcending theories about social interaction” (Svennevig, 2014, p. 306; Linell, 2009; see also Koskinen, 2022).

**Figure 1:**
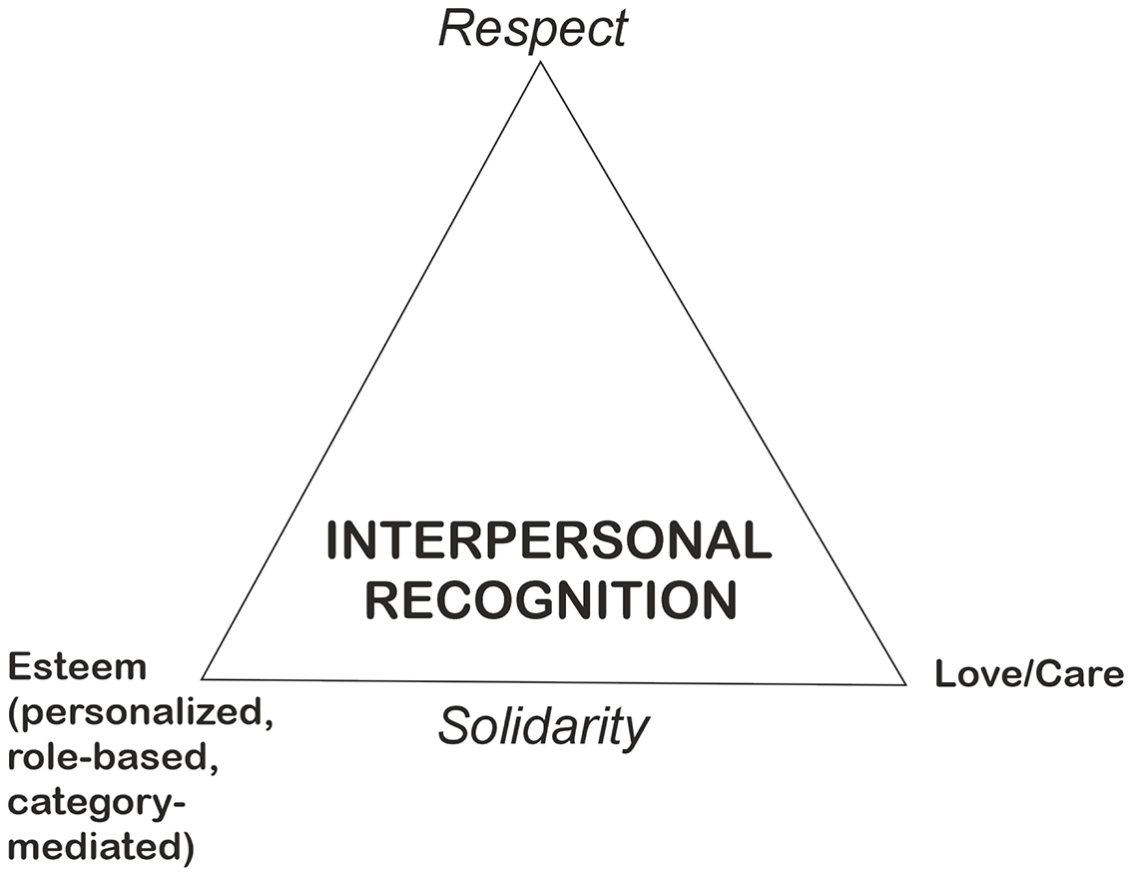
The ‘recognition triangle’ depicting the three modes of recognition: respect, esteem, and love/care.

In the current paper, we combine the theoretical ideas concerning interpersonal recognition to empirical conversation analytic observations. We operate on a model of recognition that consists of two or three different modes of recognition, depending on how fine-grained distinctions are used (*cf.* Honneth, 1995; Taylor, 1995; Laitinen, 2002). In this model, recognition is divided into the general dimensions of *respect* (recognition that one is a person, based on universality, and including positive responsiveness to one’s dignity and autonomy) and *solidarity* (recognition that one is a particular person, based on difference, and including positive responsiveness to one’s contributions, needs, and special relational standing). Solidarity, then, includes two distinct modes of recognition: the first is *esteem,* which can be based on personalized positive qualities (*what kind* of person one is, including one’s personal merits and talents) or socially valued roles (e.g., profession). Esteem can also be mediated based on the individual’s perceived category. The second is *love/care,* which refers to recognition as a singular, irreplaceable individual (*which* unique person one is). As a result, we may form a triangle of recognition that consists of respect (the dimension of respect), esteem and love/care (the dimension of solidarity) (see Figure 1). This is Axel Honneth’s threefold theory of recognition re-interpreted so that both esteem and love/care are central to solidarity, though Honneth links solidarity only to esteem. Here we follow Laitinen (2015). Apart from this conceptualization of solidarity as involving both the dimensions of esteem and love, we do not propose any specific view on the possible tensions, interdependencies, or priorities of the three modes of recognition (respect, esteem, love), and the symbolism of the triangle intends to depict this relative independence. Different theorists may meaningfully disagree, and our view is compatible with all major views that acknowledge these three modes of recognition (see, e.g., Hirvonen and Koskinen, 2022; Ikäheimo, 2022).

Respect regards persons *qua* persons (independently of what kind of person or which person is at stake). Both Honneth and Taylor see this as an important historical achievement, that equal respect is to granted to everyone universally and in a difference-blind manner: once the idea of equal human rights and the idea of dignity of persons is available, it would be wrong and disrespectful to regard anyone as a second-rate citizen. Seeds for such universalism can be found in Stoicism and many World Religions like Christianity, but both Honneth and Taylor think that only after the French Revolution such exceptionless equal respect has become an organizing principle or ideal in real societies - and remains to be fully realized. Before the modern divergence of universal respect and social esteem, it was expected that people in different positions of the social hierarchy are to be “honored” differently - second rate citizenship was the standard and so universal respect was not demanded. Nowadays it is an inescapable constitutive element of modern moral horizons (Honneth, 1995; Taylor, 1995). The two modes of recognition constitutive of solidarity are then to be distinguished from this general dimension of respect: esteem is sensitive to what kind of person is at stake (while attempting to be indifferent to which person is at stake – and treat like cases alike) and love is sensitive to which irreplaceable person is at stake: thanks to a special relationship, one cannot change the loved one to any other person, even exactly similar person. It is important to note that the use of solidarity here somewhat differs from the idea of social solidarity presented in conversation analytic literature (see, e.g., Heritage, 1984; Clayman, 2002), which originates from the ideas of Émile Durkheim (1933). In CA, conversational structures such as preference organization (Pomerantz, 1984) have been seen to follow the principle of maintaining solidarity between the members of a society. The structures are considered as universal and not difference-based: the principle holds no matter which or what kind of person you are interacting with. In this sense, CA as a theory and method has perhaps been better equipped to focus on the universal aspects of recognition than on the difference-based modes of esteem and love/care. This study aims to fill this research gap.

With all of the above in mind, we can now formulate our three research questions:

RQ1: How can we grasp recognition as an interactional phenomenon?

RQ2: How do the three modes of recognition (respect, esteem, love/care) show in interaction, either implicitly or explicitly?

RQ3: How do conversational actions operate in relation to the (mis)recognition that they convey?

In the following, we will go through the three modes of recognition in more detail. We will begin each section by describing the mode of recognition in question, and follow the general description with two empirical data examples. We utilize the structural analytical framework of CA to investigate how interpersonal recognition happens in and through sequences of social interaction when one person seeks to attain a status of a ratified interaction partner (respect), seeks acknowledgement for their individualized and/or category-based characteristics, and/or invites, and makes themselves relevant for, others’ love and care. A key principle in CA is that various features of the delivery of talk and other bodily conduct are crucial to how interlocutors build specific actions and respond to the actions of others (Hepburn and Bolden, 2013, p. 57). This is why we utilize transcripts that include the details of how something is said, based on the assumption that “no order of detail in interaction can be dismissed *a priori* as disorderly, accidental, or irrelevant” (Heritage, 1984, p. 241). We provide a three-line transcription, where the first line represents the original talk, the second line is a morpheme-by-morpheme English gloss of the original, and the third line is an idiomatic English translation (cf. Hepburn and Bolden, 2013, p. 68–69). The advantage of a three-line transcription is that it allows an understanding of the talk as it temporally unfolds. See Supplementary material for explanations of the transcription symbols and glossing abbreviations.

The data extracts will illuminate how recognition shows in interaction through cases of momentary misrecognition. We use ‘misrecognition’ as a general term for missing, incomplete and/or wrong kind of recognition. We examine data from various conversational settings (institutional, quasi-experimental, family interaction) and activities (decision-making, storytelling). At the end of the article, we discuss the implications of the presented results for the interface of conversation analysis and sociological theory.

## 2 Respect

Respect is in principle owed to all persons equally just because they are persons: autonomous, rational, moral agents capable of leading their own lives and taking part in collective decision-making. The mere fact *that* one is a person thus suffices to ground demands of rights to be respected as such. Once these rights are violated, experiences of disrespect are a typical and fitting response. Clearest violations of equal respect may be ones that are encoded in the structures of society: for example a caste society can officially regard some people as superior and others as inferior. In modern societies, the aim (not always successfully realized) is to guarantee everyone an equal position. *Interpersonal* violations of respect show up in acts and attitudes of individuals, and it is those violations that are of interest for researchers of social interaction.

As an attitude, genuine respect is based on the acknowledgment of the equality and dignity of the other. Respect can be characterized by reverence and the maintenance of distance, instead of lovingly rushing to help the other to lead their lives. By contrast, disrespectful treatment can vary from blatant violations of rights to subtle nuances of expressions, say, mere tones of voices, when things that are justifiable as such are said (or done) in disrespectful ways. Anything can be done disrespectfully, if accompanied with disrespectful expressions or attitudes (See, e.g., Thompson, 2006; McBride, 2014; Dillon, 2022; Ikäheimo, 2022; Siep et al., 2022).

Extract 1 shows an example of misrecognition in the sense of respect. The case is drawn from a co-development workshop, in which professionals in a large social and healthcare organization and the so-called experts-by-experience discuss ways in which the delivery of the social and health care services could be improved (see Weiste et al., 2022). In this case, the participants have previously discussed how to collect feedback from the clients. Just previously, one of the professionals has proposed that the feedback be collected via email, which is also referred to by her colleague (P1) at the beginning of the extract (l. 1–4). At this point, one of the experts-by-experience (E1) takes a turn in the discussion, telling a story about the way in which she has previously helped a mother who had felt that his son had been unjustifiably excluded from the services.


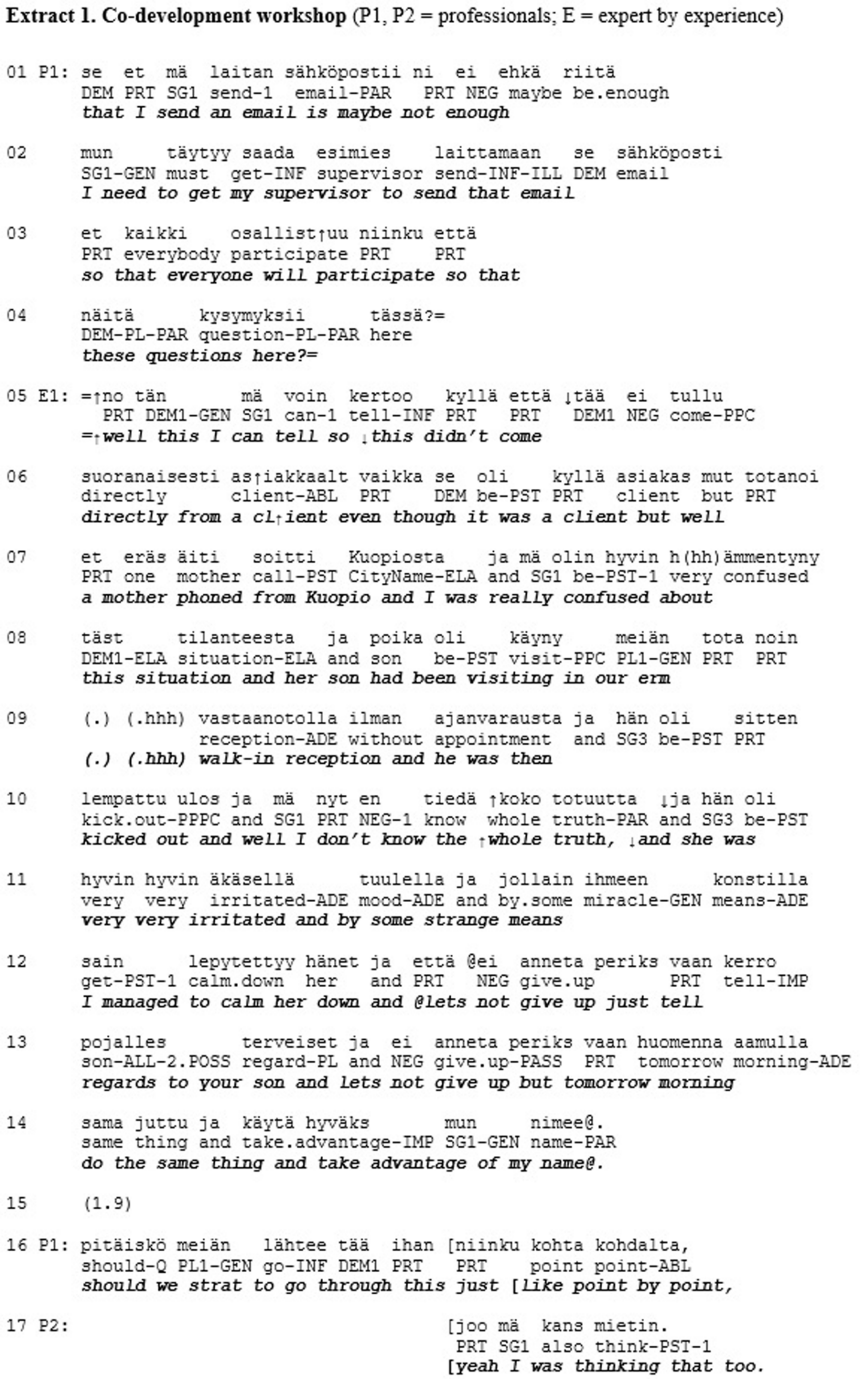


As a response to the proposal on the feedback collection method, E1 tells about a particular situation in which she received negative feedback and describes how she handled the situation (l. 5–14). During the telling, the other participants produce no response particles (e.g., “okay”), that would encourage E1 to continue her telling or display that they are listening (Sorjonen, 2001). Upon the completion of E1’s account, all story-recipients remain silent (l. 15). Next, the professional returns to the workshop agenda and, by referring to the assignment sheet, proposes that the participants begin working through it (l. 16). The other professional agrees, stating that this was something that she was also considering (l. 17). Thus, the story here is ‘sequentially deleted’—that is, completely ignored by the professionals (see Weiste et al., 2022, p. 12).

As stated above, respect refers to the universal recognition that one is a person. Persons are those “toward whom other persons take a ‘personal stance’, or whom others relate to as ‘respondents’” (Laitinen, 2002, p. 474). To be an actual person means to be taken by others as having the right to be respected as a person, and, for example, refraining from treating someone as a responsible agent and a communication partner involves a violation of such respect. In our view, Extract 1 depicts a micro-moment of interaction, where the expert-by-experience (E1) is *not* treated as an equal communication partner but someone whose views can be considered as irrelevant and not worthy of even minimal acknowledgment. Hence, the extract depicts a momentary lack of respect in recognition theoretical terms.

Let us consider another example of how respect—as the fundamental category of recognition—can be at stake in social interaction. Extract 2 is drawn from a study by Valkeapää et al. (2019) and represents a situation that is explicitly framed as being about joint decision making. The decision making takes place within a Clubhouse community—a non-profit organization providing mental health rehabilitation based on membership in the community (Raeburn et al., 2013). The Clubhouse members who wish to enter the labor market are supported by the Clubhouse-created transition employment programme, which involves part-time short-term employment at various cooperating companies. The selection of the individuals getting the chance to try transitional employment is managed by the Clubhouse community, not by the employers. Once succeeded in transitional employment the Clubhouse members have better prospects to seek competitive employment (Henry et al., 2001). The decisions about entrance into transitional employment are thus highly consequential to the Clubhouse members, which is why it is explicitly stated that these decisions should be made democratically in the community (Valkeapää et al., 2019). Extract 2 is from such a decision-making situation.


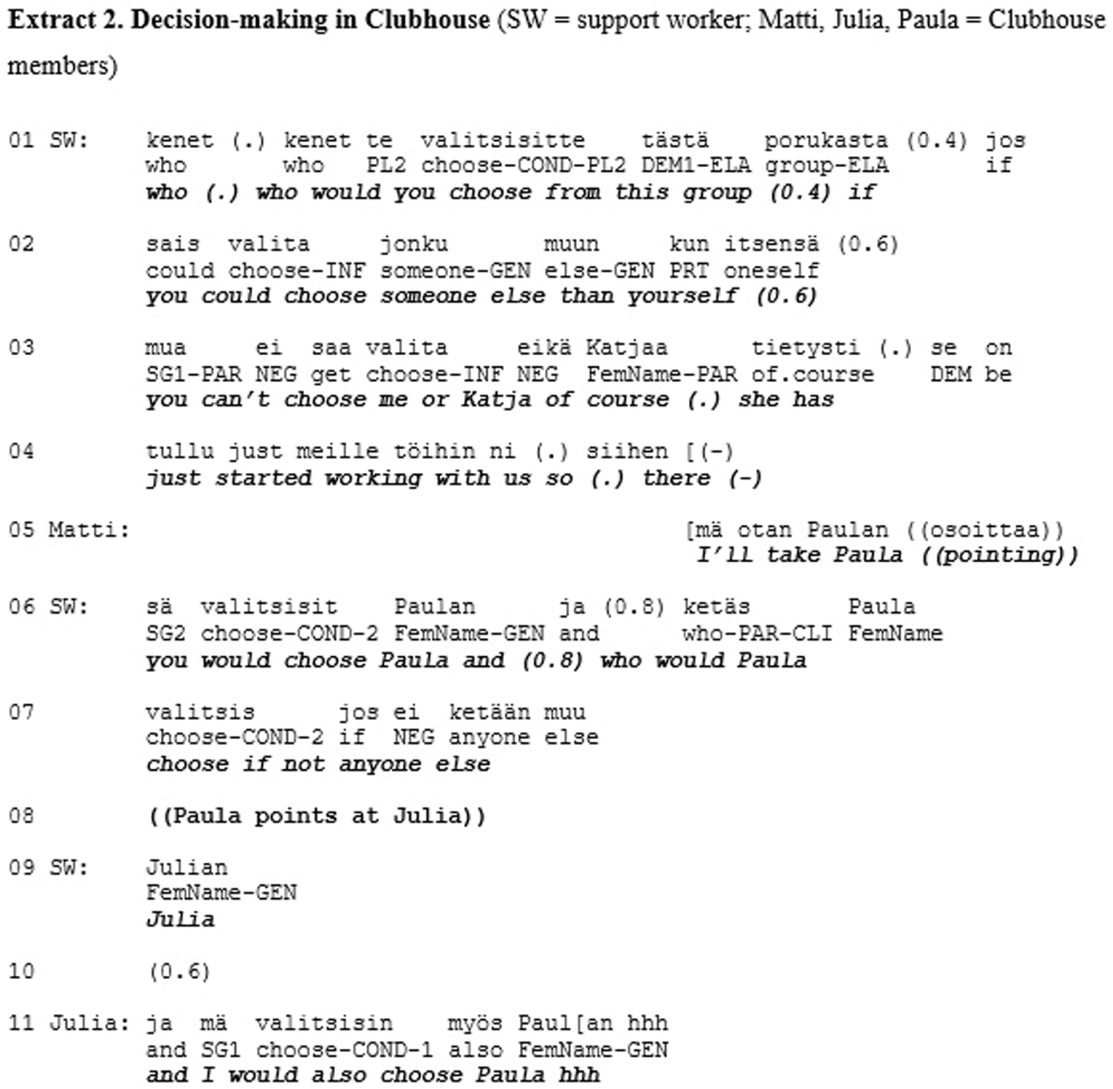



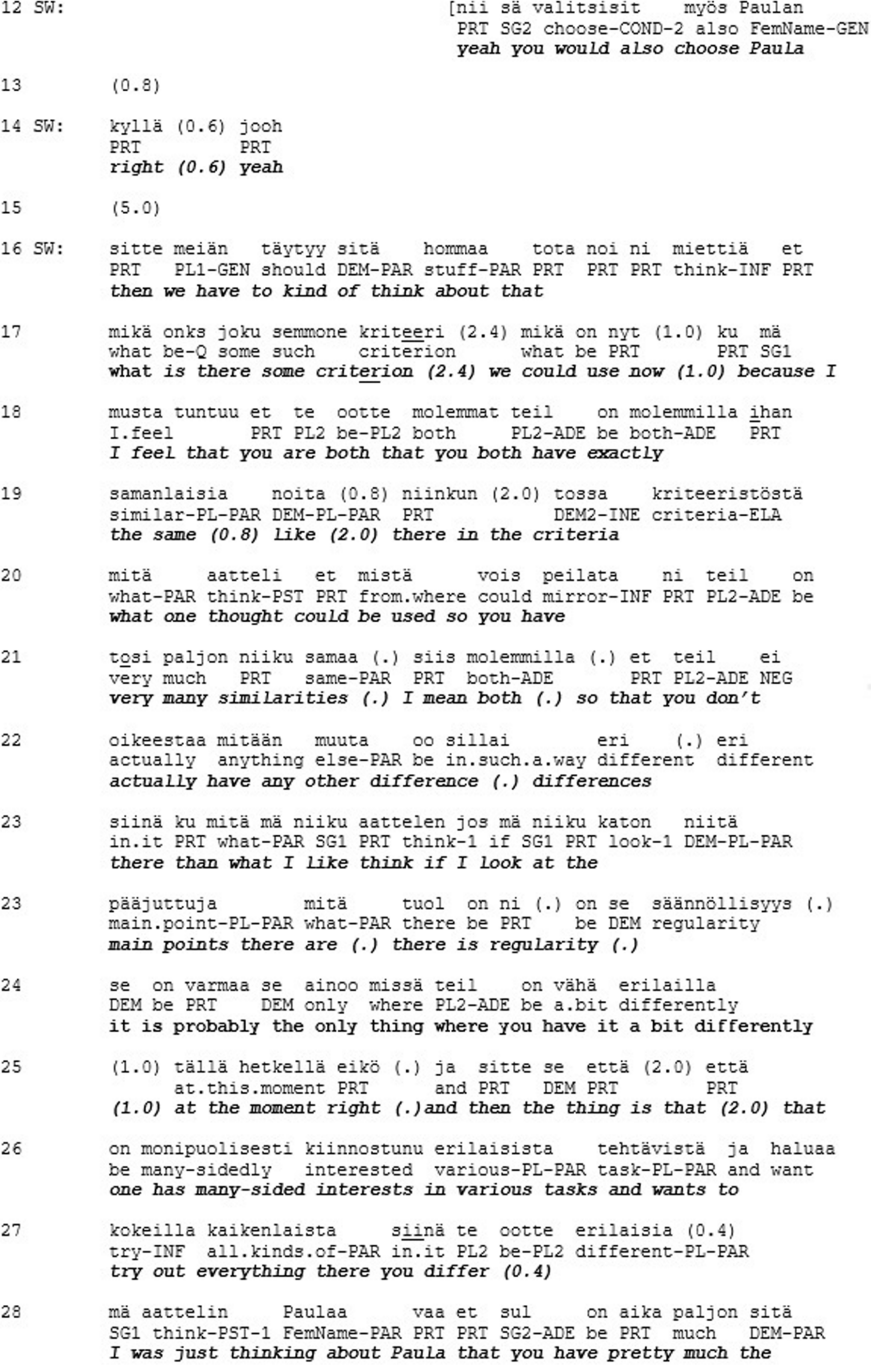



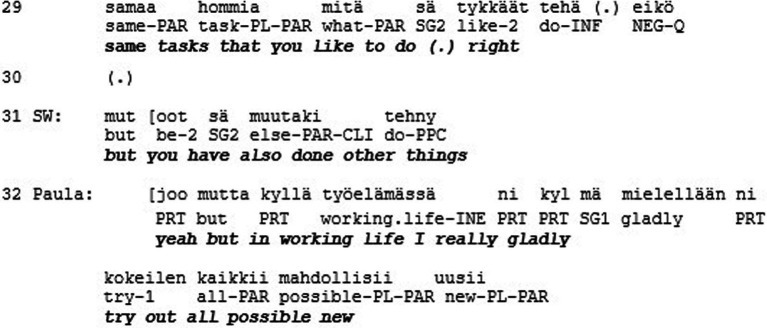


At the beginning of the extract the support worker (SW) initiates a procedure to get the Clubhouse members vote among themselves the one who they think should get the employment (l. 1–4). The voting procedure results in Paula getting most of the votes (l. 5–14). However, instead of declaring Paula the winner of the vote, a long silence follows (l. 15). Thereafter the support worker announces a next item in the agenda, which is to come up with the decisive criterion for making the decision (l. 16–17). In so doing, the support worker effectively undermines the relevance of the previous voting result. Instead, a much more unilateral way of making a decision is reflected in the support worker’s lengthy evaluative account of the qualities of the candidates (l. 16–29). In essence, the support worker introduces ‘regularity’ and ‘versatility’ as criteria with reference to which Paula and Julia are said to differ (l. 21–27) and Paula to fall short (l. 28–29). Thereafter, in an attempt to request for Paula’s confirmation for his assessment, the support worker uses a turn-final question particle (*eikö* ‘right’ l. 21). However, most likely in anticipation of an upcoming disagreement, the support worker softens the assessment, while Paula indeed produces a disagreement in overlap with the support worker. In displaying a need to defend herself against the criteria imposed by the support worker (l. 32–33) Paula orients to the possibility that she may still lose the selection, irrespective of her just previously having won the vote. Thereafter the support worker directs the selection process in a way that it really is Julia, and not Paula, who will be selected to get transitional employment.

Hence, the participants in the encounter were given the possibility to express their opinion in the matter that was of great significance to them. Unlike in Extract 1, the participants were explicitly asked about their views and their answers were minimally acknowledged as received. However, acknowledging a person as a conversational participant is not necessarily enough to convey respect. Here, we may observe a lack of respect, which shows in the lack of consequentiality that some participants’ interactional contributions have for the overall joint activity. In this case, the consequences were not only about influencing the trajectory of the interaction in the here and now of the encounter, but also about the participants’ lives beyond and after the encounter. However, the role of the “responsible agent” (Laitinen, 2002, p. 474) who may participate in decision-making about these consequences was withheld from these participants.

## 3 Esteem

Esteem as a type of recognition focuses on the person’s particular traits, achievements, merits, laudable efforts, talents, contributions, admirable features, and so on, that are different with different people. Yet, esteem is ideally indifferent toward (numerical) identities in the sense that the same praise is adequate for the same efforts, talents, contributions independently of who (say, who’s nephew or neighbor) is in question—various norms of impartiality forbidding nepotism are embodied in a number of practices from anonymous peer review to public announcements of conflicts of interest in recruitment.

Esteem comes in different variants. What holds all forms of esteem together is that they are positive feedback on one’s qualities or features that are typically different with different people. The most straightforward case of esteem is based on one’s achievements or actions: doing one’s job well is a basis for positive appraisal by others. On top of the kind of esteem that everyone holding a role of the same kind may share (see below), there is *personalized* esteem that consists in the feedback and attitudes of others concerning how well one is doing one’s job. So different teachers are esteemed to different degrees, because of differences in the style and effects of how they live and perform in that role. Personalized esteem is not restricted to how well one performs in such central defining roles as in one’s job—one can be held a valuable contributor to social life merely by being a fun person to hang out with. Having valuable personal features from admirable character traits to exceptional talents or to good looks may be a basis of personalized esteem.

In addition to such personalized feedback, the socially recognized *role* one occupies can as such be a source of esteem or esteemworthiness: think of being a teacher, priest, president, garbage collector or professor. Different jobs, offices or roles come with a certain type of social standing in the eyes of others—being a professor comes with some amount of default esteem. That is something that all people in that role share, independently of how well they do that task. Often one’s salary is dependent on what the title of one’s office or role is (perhaps combined with personalized bonuses dependent on one’s actual performance). Typically, recognizing someone’s role also shows in being treated as an expert on questions related to that role.

Further, being perceived as belonging to some special categories or groups may lower (or heighten) others’ expectations and assessments of one’s performance. For example, being diagnosed or perceived with some permanent or temporary condition may be thought to affect one’s performance in ways that lower the expectations. When one is known to be sick, one is not expected to carry on with one’s tasks as usual. The mere fact that one has the condition does not lower the expectations, the interaction partners must perceive or assume or know or mistakenly confer such category-membership on the individual (Ásta, 2018). These category-memberships may not as such carry special positive esteem with them - and indeed, expressing lowered expectations may be experienced as disrespectful - but they may nonetheless meaningfully and positively affect how individual achievements are assessed and esteemed: what for many others might seem as average performance, can be an achievement for someone categorized as having extra challenges. Below we call esteem mediated by perceived membership in categories that come with differing expectations *category-mediated esteem.*

Extract 3 shows an example of misrecognition in the sense of esteem. The extract is from a quasi-natural dataset where individuals diagnosed with Asperger syndrome (AS) discuss with neurotypical (i.e., persons without neurological diagnoses; NT) individuals about their personal lives (see Koskinen, 2022). The two male research participants sit in armchairs facing each other perpendicularly. They have been asked to talk about happy events and the losses in their lives in a freely chosen way. In the following extract, the AS-participant (T) tells the NT-recipient (R) about one of his successes in life, which is that he graduated from high school on his very first try.


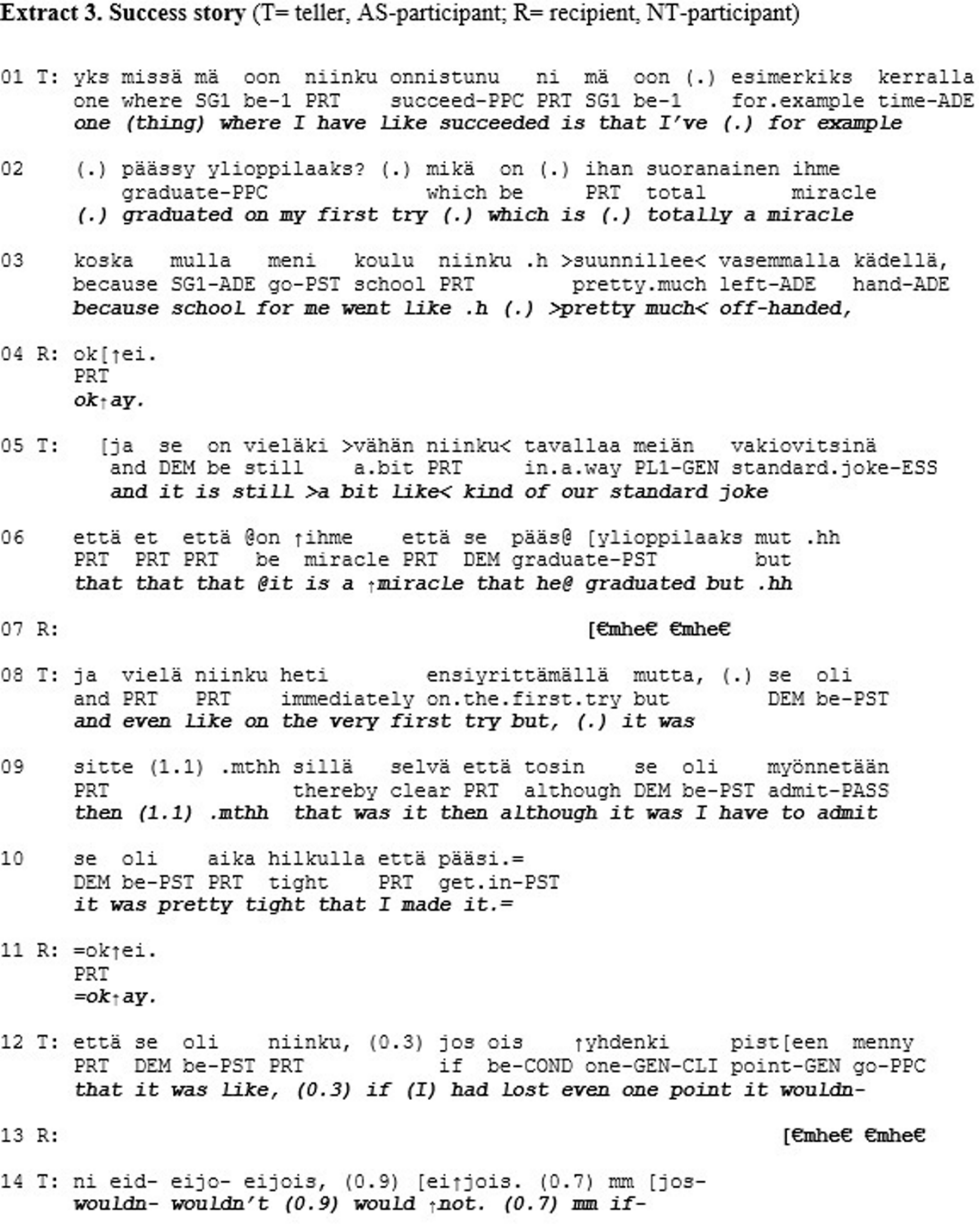



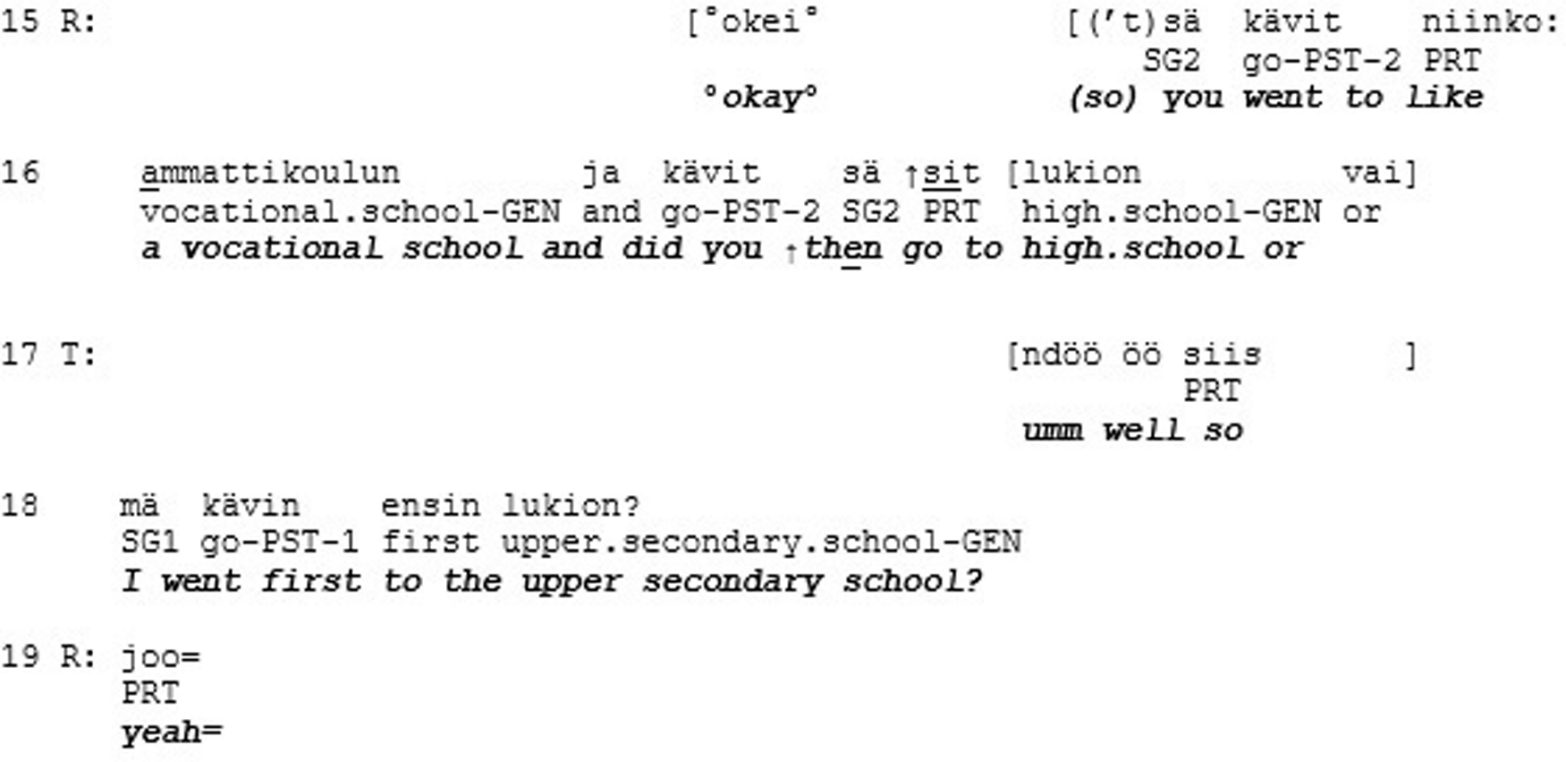


The teller (T) first makes the positive affective stance of the telling (cf. Stivers, 2008) explicit by mentioning that this is a thing where he has succeeded (l. 1). He continues by declaring that he graduated on his first try (l. 1–2). This, however, only elicits a slow, upward nod from R, which leads T to continue “which is totally a miracle because school for me went pretty much off-handed” (l. 2–3). This elaboration elicits a minimal response “okay” from R with a slightly surprised prosody (l. 4). Then T continues by slightly changing the affective stance by making it humorous “and it is still kind of our standard joke” and imitates direct reported speech “it is a miracle that he graduated” (l. 5–6), which receives a small laugh-token from R (l. 7). The lukewarm display of affiliation by R is understandable, as self-deprecating stances can be difficult for recipients to endorse (Pomerantz, 1984). Then T returns to his original stance of success by emphasizing “and even like on the very first try” (l. 8), which does not elicit any reaction R. Then T continues “although I have to admit that it was close” (l. 9–10). This, again, only gets an acknowledgement “okay” from R (l. 11). Then, finally, after T has emphasized again how close his graduation was (l. 12, 14), R responds to the telling with a longer turn: “(so) you went to a vocational school and did you then go to upper secondary school or” (l. 15–16). This question clearly circumvents the stance of success that T has introduced in his telling (cf. Koskinen et al., 2021). After the question, the participants continue to talk about T’s school background and neither the feeling of success nor the event of graduating is returned to.

As stated above, esteem is based on the different (positive) qualities, merits, and achievements that individuals have. In Extract 3, the AS-participant does *not* receive this type of recognition for his achievements, even though the telling would have made such recognition relevant. As mentioned in the previous paragraph, however, tellings with self-deprecating stances are difficult to affiliate with. In the same way as story-recipients may sometimes refrain from empathetic turns in order to save face of the teller, it could be considered face-threatening to show enthusiastic affiliation (e.g., “that is amazing”) to someone succeeding in a ‘standard performance’, as graduating from high school could be culturally considered to be. With his question, R reframes the topic under discussion to be a broader one of studies, as opposed to the more specific event of T’s graduation, and in this way manages to bypass the possibly face-threatening moment. The other-attentiveness of the question can also work to legitimate the topic shift (*cf.* Jefferson, 1984; Koskinen et al., 2021).

We argue that the recipient here is oriented to personalized positive qualities of the teller in relation to other people, instead of category-mediated esteem. Meaning that R assessed the personal merit of T’s graduation in relation to the overall percentage of Finnish high school students who graduate on their first try (which is about 94%). Making this comparison, graduating on the first try could be deemed not-so-impressive, and showing too great appreciation for that achievement could be interpreted as patronizing. However, the other route would have been to assess T’s performance in relation to his being an individual on the autism spectrum. One of the hallmarks of Asperger syndrome is the AS-individuals’ uneven cognitive profiles (e.g., Attwood, 1998), which can cause their academic success to suffer. Against this backdrop, T’s success story would indeed invite strong affiliation from the recipient, which it does not receive. Hence, the extract depicts a momentary lack of (category-mediated) esteem. The recipient had a chance to signal being on the same side, show solidarity, and regard as salient those criteria of esteem that would have allowed the sense in which it was a genuine achievement to show. What was a successful bypassing of a face-threatening moment from one angle, was from another angle a failure to show esteem that would have been appropriate (The phenomenon of describing or redescribing a situation so that the other can appear in positive light is relevant to the ethics of Iris Murdoch, see The Sovereignty of Good (Murdoch, 1970), and is arguably central for standing in relations of solidarity, for “being on someone’s side.” For an overview on the notion of solidarity, see Sangiovanni and Viehoff, 2023, and for its relationship with recognition, see Laitinen, 2015).

We will now move on to analyze a case in which two components of esteem*—*the one based on role-specific status and the one based on positive personalized qualities—both serve as possible bases of positive assessment. Extract 4 is drawn from the study by Stevanovic and Peräkylä (2014). Here, a pastor (P) and a cantor (C) discuss the Pentecost mass, and the cantor shows to the pastor a hymn arrangement that he has made for the event (l. 1–2).


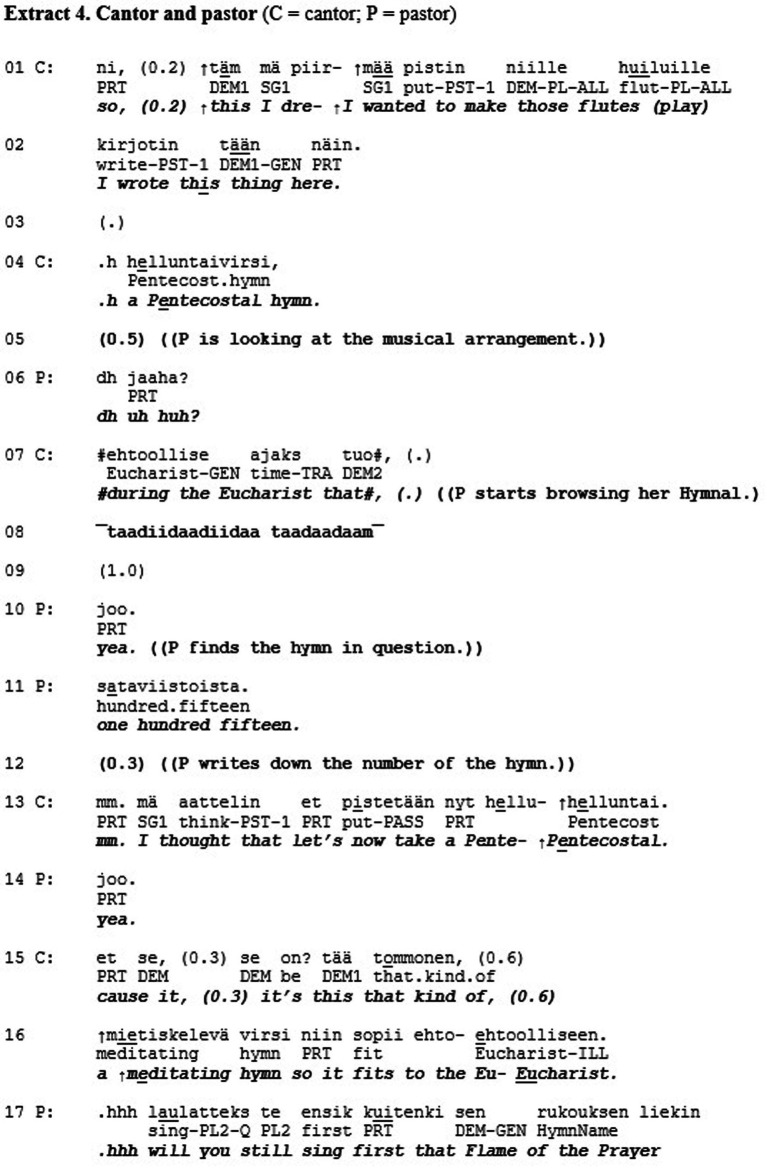


The cantor’s utterance is an announcement of a decision, which calls for the pastor’s acceptance of it (l. 1–4). However, by showing the arrangement to the pastor, the cantor can also be heard as inviting an assessment by the recipient. The utterance is designed in a way that foregrounds his own role as the creator of the arrangement, which invites the pastor to express her appreciation for the cantor’s accomplishment. These two options—a display of acceptance of the cantor’s decision and an assessment of the cantor’s accomplishment—are thus both potentially relevant ways of responding to the cantor’s line of action. The first one would provide recognition of the cantor as one who, by virtue of his professional role, has the “deontic authority” (Stevanovic and Peräkylä, 2012) with reference to the realization of the Eucharist hymn in the upcoming mass. The second one, in contrast, would provide personalized recognition of the cantor as a specific *kind* of representative of his profession—one who has invested an exceptional amount of work in the preparation of the mass and, in so doing, accomplished something extraordinary.

The pastor chooses to pursue the first option: she treats the cantor’s utterance as an announcement of a decision. The pastor receives this information first by checking the arrangement (l. 5), then by starting to leaf through her hymnal to find the hymn (l. 7), and, after having found the hymn (l. 10), by writing down its number (l. 12). In and through all this, the pastor displays commitment to treating the cantor’s hymn choice as binding. The cantor, however, does not seem to treat the pastor’s responses as sufficient. This can be seen in the ways in which the cantor starts to account for his choice of music, explicating the grounds for his decision (l. 13, 15–16). In so doing, the cantor invites the pastor to display appreciation of his choice of music. Importantly, as both participants have displayed an orientation to the decision as established, the cantor is not asking the pastor to participate in the decision-making as such (Stevanovic, 2012). Rather, he invites the pastor to recognize that the cantor has fulfilled her professional role in a specifically applaudable manner. The pastor, however, refrains from providing such personalized recognition. Instead, she asks the cantor about the order of the musical items in the mass (l. 17), thus sticking “strictly to business,” which involves recognition of the cantor’s role-specific status as the sole decision-maker in the matter at hand. Yet, it constitutes a failure to give personalized feedback for an achievement when it would have been appropriate; a misrecognition in the mode of esteem.

## 4 Love/care

Love or care is the third main form of recognition. Two ways of distinguishing it from respect or esteem are worth mentioning. The “logic” of love is not difference-blind either in the sense of universal respect of generalized others, or impartial esteem conditional on one’s qualities. Love is a way of regarding the significant other as irreplaceable, a special, singled out and unique person. Love need not be deserved, and it is not conditional on achievements like esteem. Further, the “ground” of loving care seems to be something like the vulnerability and neediness of the other, the capacity to feel not only positive emotions but also to suffer, rather than their autonomy, merits, or roles. The variants of human relationships that are constituted by such recognition of vulnerability range from parental and romantic love to friendship, and in wider circles, solidarity. Solidarity can be seen as a combination of mutual esteem and mutual care, where each party is potentially a beneficiary of support from others, and a supporter of others. Love, care, friendship and solidarity show up in ways of treating the other but also in one’s own emotional responses regarding the other; and typically third persons can modify their expectations and take into account the parental or romantic or otherwise significant relationships and friendships of others.

Extract 5 shows an example of a misrecognition in the sense of love/care. The extract is from a quasi-natural dataset where individuals with a high level of narcissistic personality traits (N+) discuss with individuals who have low levels of these traits (N-, see Koskinen et al., *in review*). The two female research participants sit in armchairs facing each other perpendicularly. In this segment, they have been instructed to tell about moments where they felt ashamed of themselves. In the following extract, the N- participant (B) tells the N+ participant (A) about an incident with her PhD supervisor that caused her to feel ashamed.


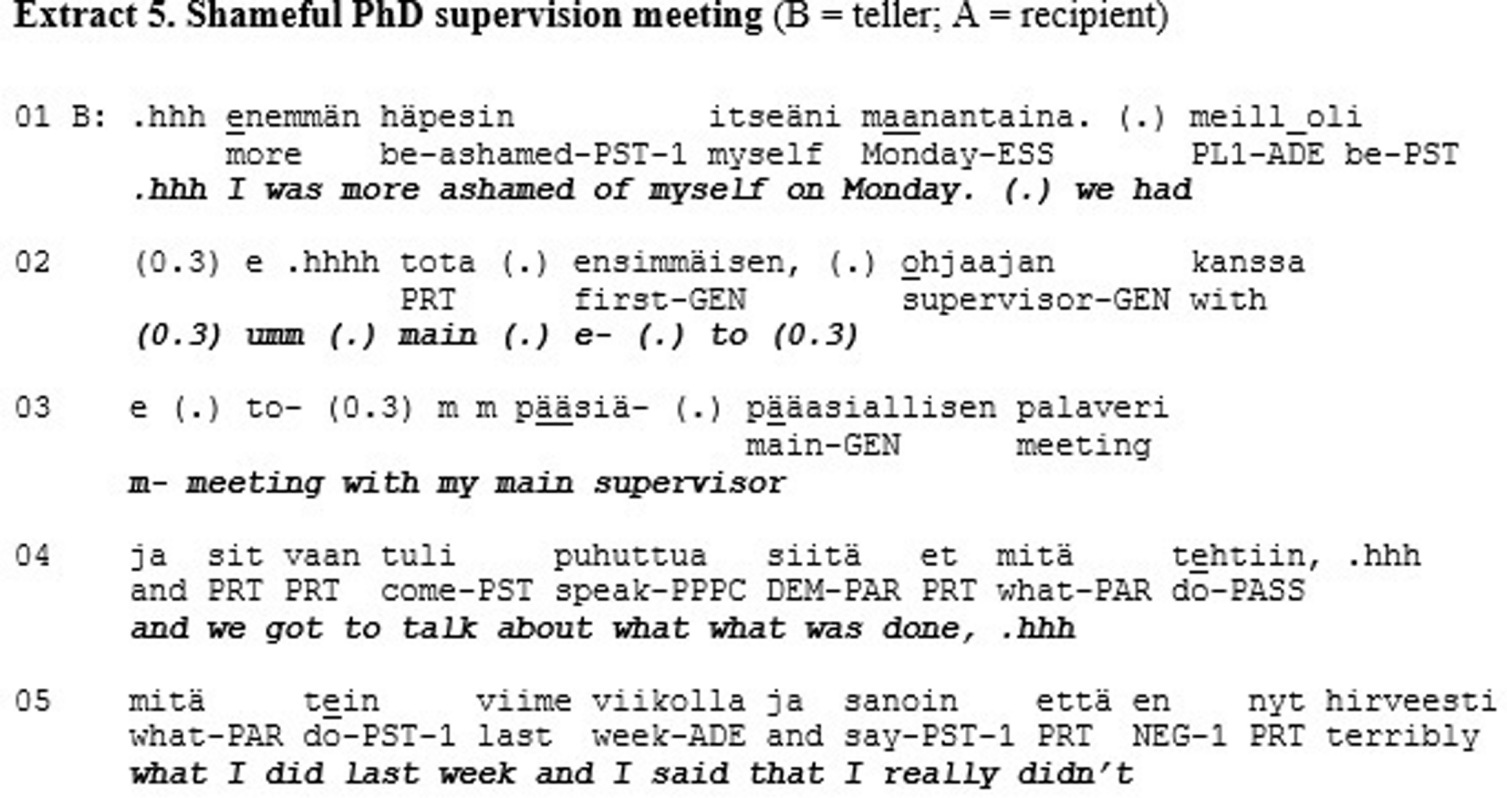



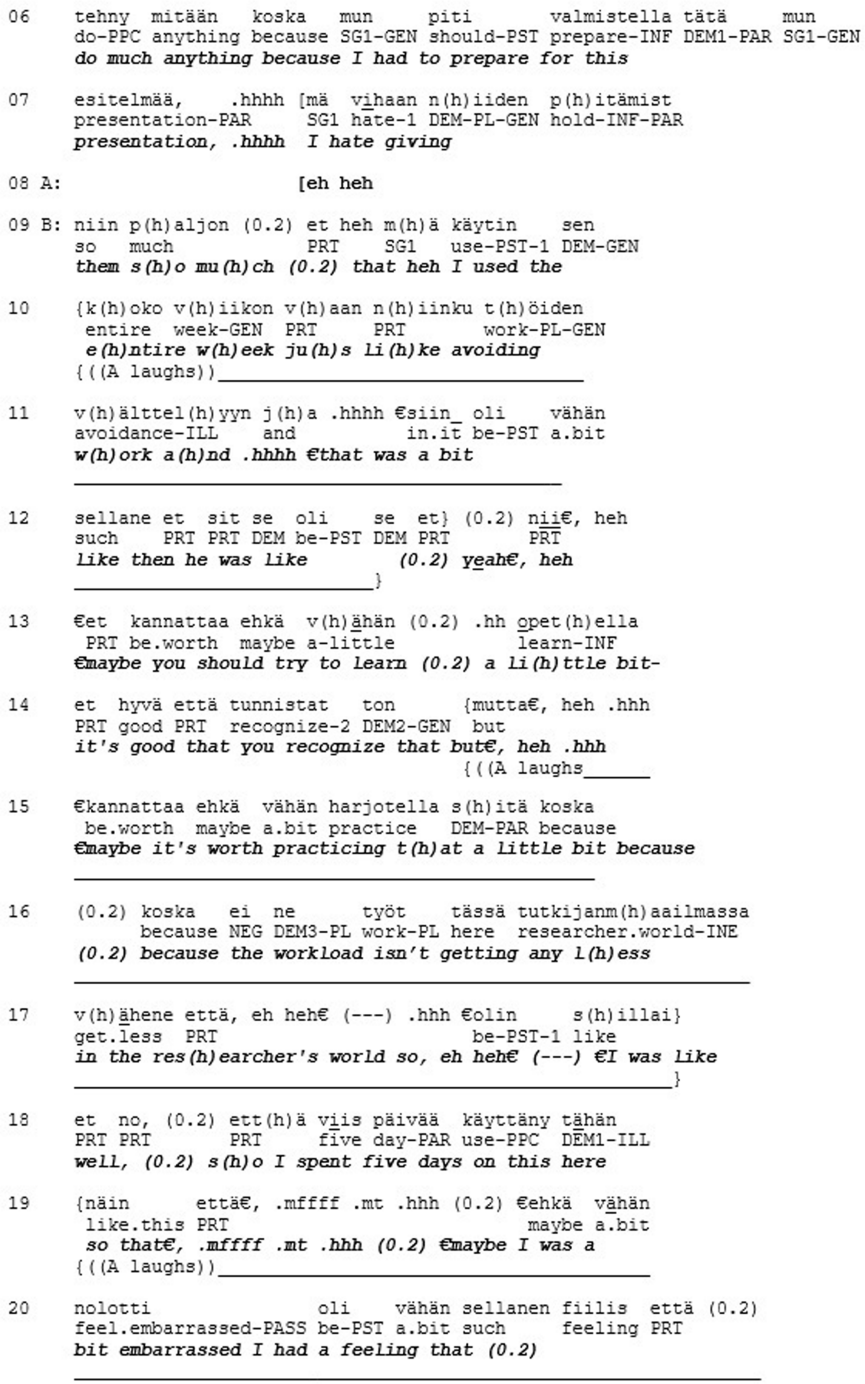



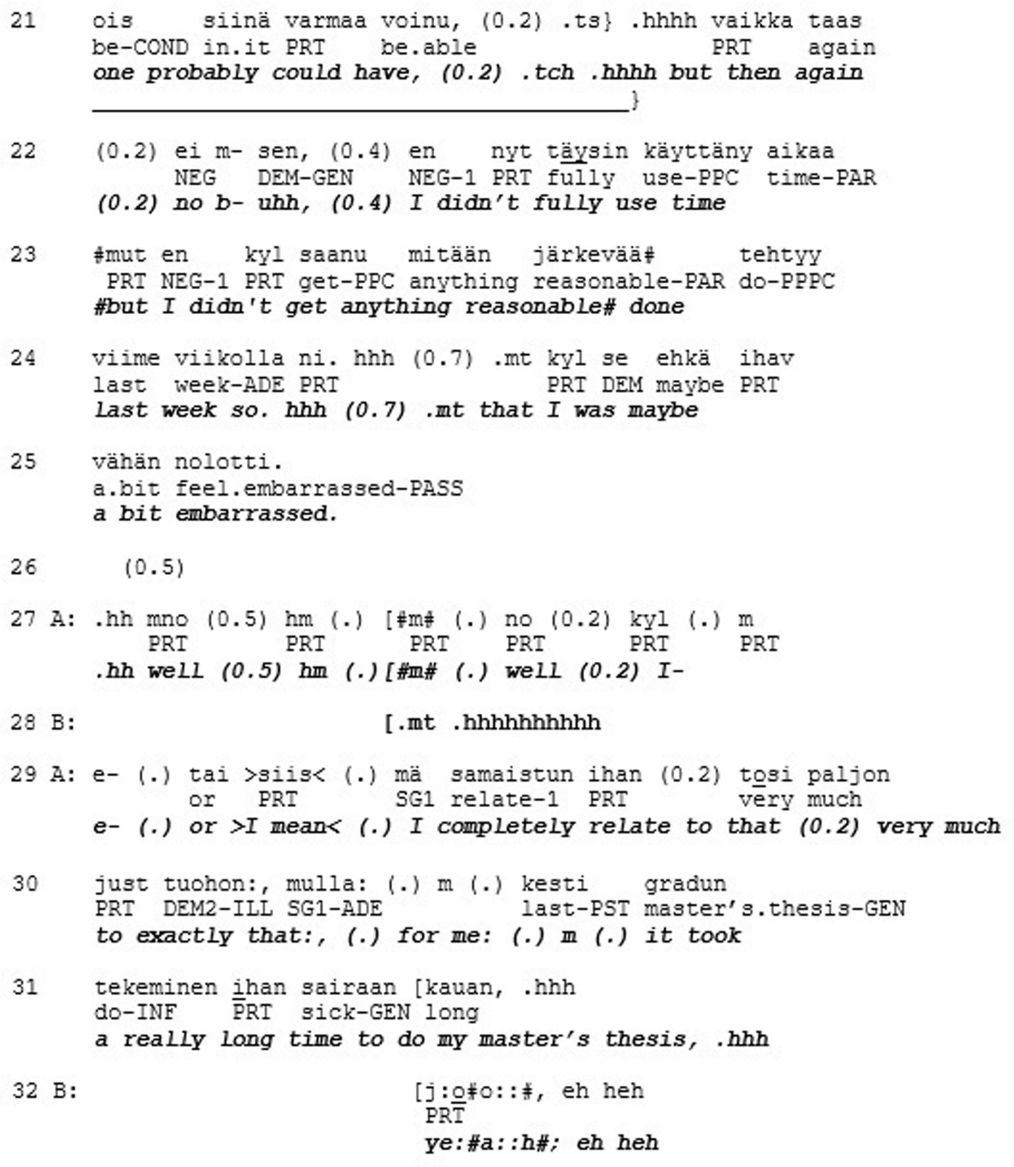


The teller (B) describes a meeting with her supervisor where she admitted not getting anything done in the previous week (l. 1–25). The story is produced with a humorous, laughing tone, and the recipient (A) produces affiliative feedback (i.e., smiling, laughter) throughout the whole telling (l. 8–21). After B is finished with her story, A displays affiliation with “I completely relate to that, very much to exactly that” (l. 29–30) and produces a short second story (Sacks, 1992) about her own masters thesis that took a long time to do, affiliating with A by conveying to her ‘I’m with you’ (see Jefferson, 2002).

The recipient here shows recognition toward the teller’s emotional experience and responds to the story’s evaluative properties in an adequate way. This is not only recognition of B as a person (respect) but also recognition of what kind of person B is (esteem): the recipient is accepting and validating these special features, publicly relating to them. What then could be missing here in terms of recognition? The second component of solidarity (love/care) involves recognizing someone as a singular, irreplaceable individual. When A displays affiliation toward B by sharing the experience and emphasizing the similarity between them, the uniqueness of B and her experience actually gets lost in the process (cf. Heritage, 2011). A could have recognized the ‘vulnerability and neediness of the other, the capacity to feel not only positive emotions but also to suffer’ (see above). Response of this kind could have, for example, applauded B’s courage in giving a presentation, even though she hates them, or admired her honesty and vulnerability in divulging this shameful incident. This level of recognition, however, is most likely less common in conversations between previously unacquainted individuals. It could nonetheless be utilized here, and without the theoretical tools of the three different modes of recognition, this aspect would stay hidden and inaccessible for analysis.

The previous extract was an example of recognition on the level of esteem but not love/care. This final extract is an example of the opposite: recognition on the level of love/care but not esteem. Furthermore, Extract 6 deepens our understanding of the mode of love/care by showing how recognition can operate on a different level and independently from conversational actions. The extract is from the conversation data archive at the Department of Finnish, Finno-Ugric and Scandinavian Studies at the University of Helsinki (Sg441). The segment is from an naturalistic everyday interaction on a family dinner between Jorma (father), Virpi (mother), Liisa (daughter) and Jarkko (daughter’s boyfriend). Here the family members are finishing up their dinner when the mother (Virpi) brings up a plan of painting a box with chalk paint, so that they could write things on the box.


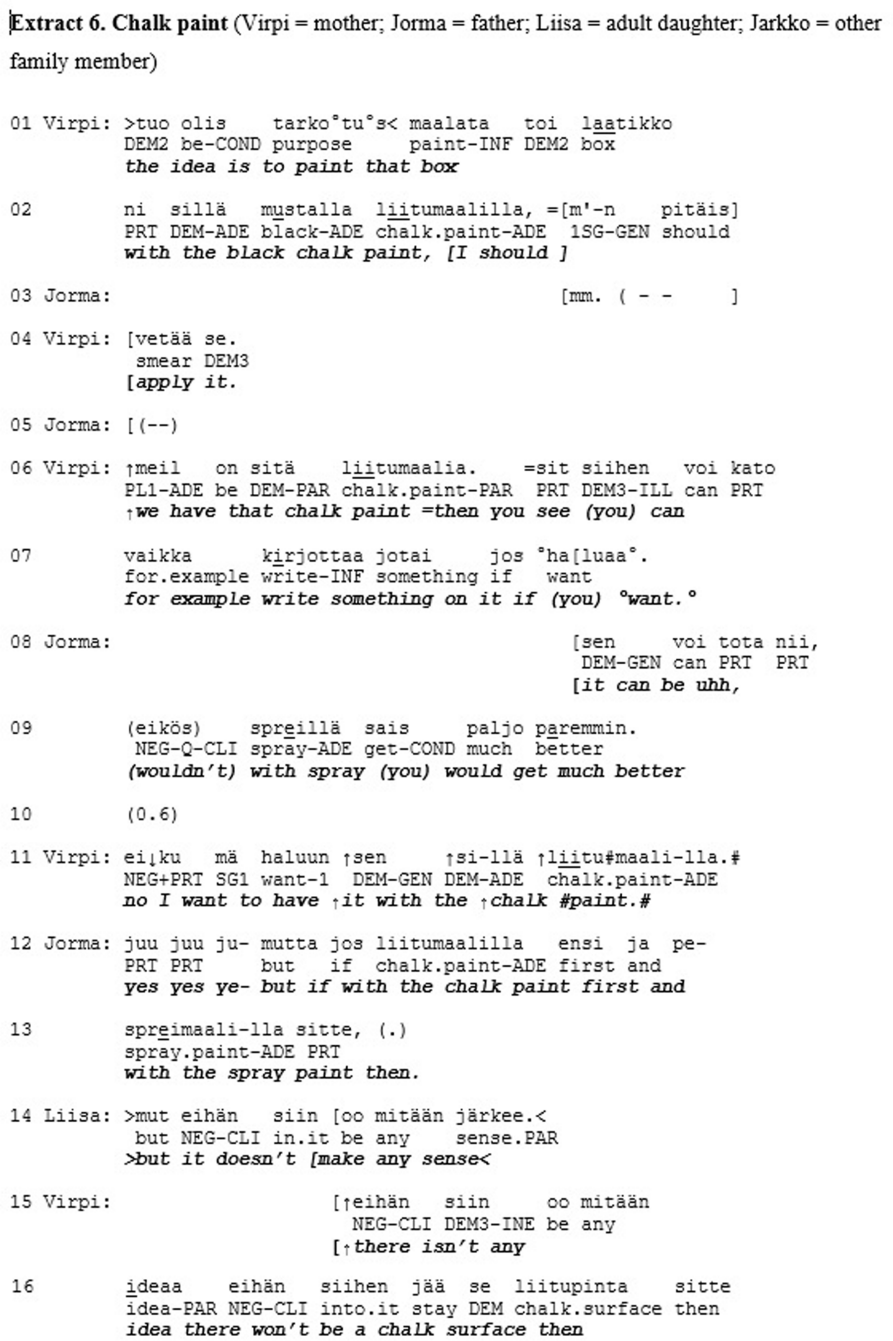



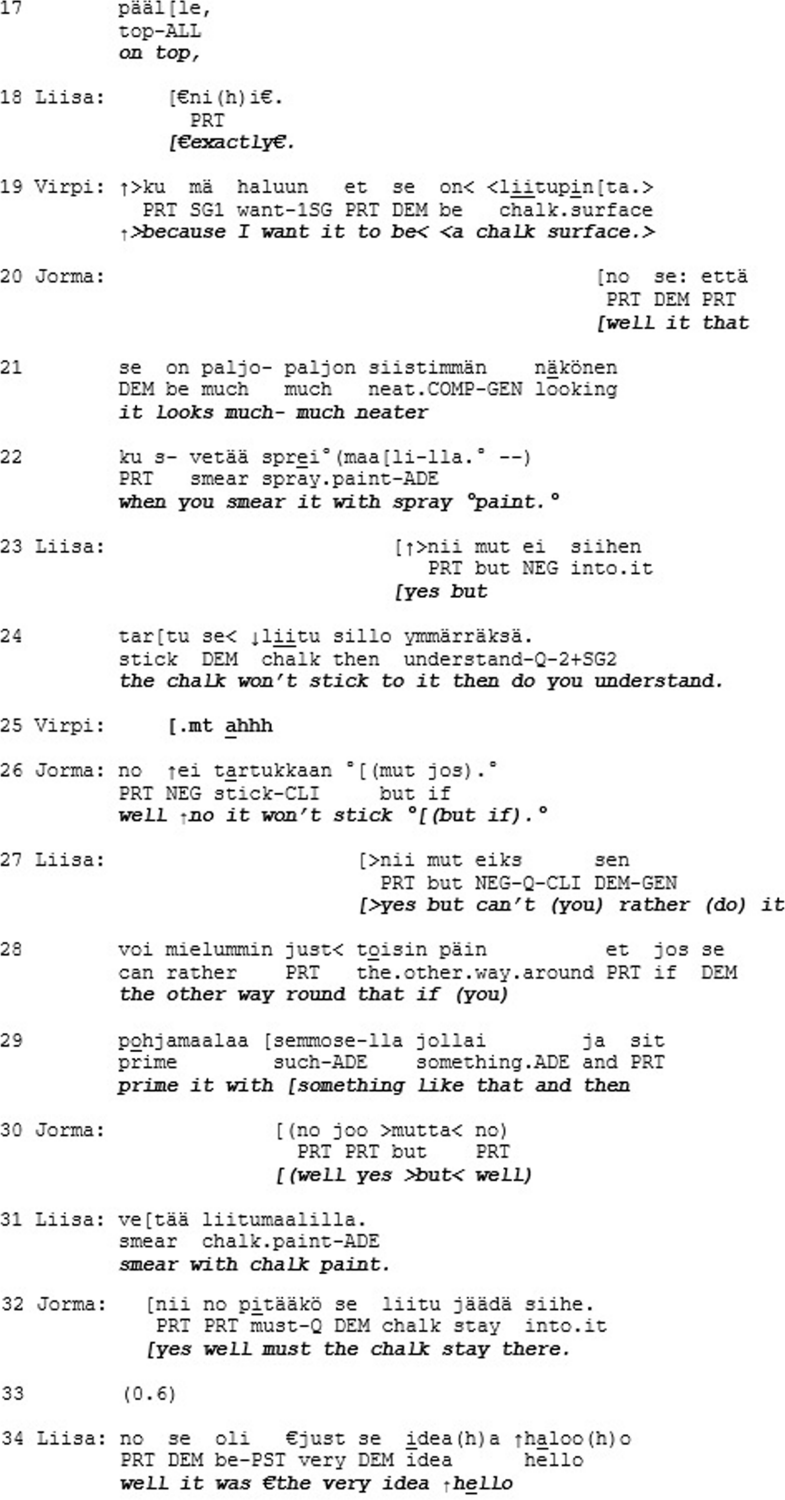



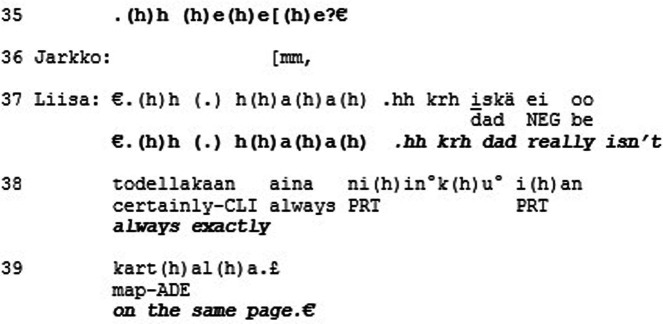


Virpi makes the announcement to the other family members that her idea is to paint the box with black chalk paint (l. 1–2). Jorma’s response is quiet and indeterminable (l. 3, 5), and Virpi continues by clarifying that they already have the paint available (l. 6) and explains how they could then write something on the box (l. 6–7). Jorma questions this plan and asks if spray paint would be better (l. 8–9). Virpi responds with a sing-song voice “no I want to have it with the chalk paint” (l. 11). To this Jorma suggests a compromise that they would use the chalk paint first and then spray paint over it. (l. 12–13). At this point the daughter Liisa joins the discussion to point out that this would not make any sense (l. 14), and Virpi seconds her by explicating that then the chalk surface would not be there (l. 15–17). Once more Jorma suggests that the spray paint would look better (l. 20–22), after which Liisa repeats their reasoning that then the chalk would not stick to the paint and adds “do you understand” (l. 24), emphasizing the miscommunication about the matter. They continue the discussion.

until finally Jorma asks “yes well must the chalk stay there?” (l. 32). This receives open mocking “hello” and laughter from Liisa while Virpi rolls her eyes and head (l. 34–37). Liisa concludes with a general assessment concerning Jorma: “dad really is not always exactly on the same page” (l. 37–39), to which Jorma responds quietly “what” (l. 40).

This is a case of overt disagreement and misalignment, and perhaps even misunderstanding between the family members, specifically Jorma and the others. In the mode of esteem, Jorma does not receive much accolade. He does not receive recognition for his merits, accomplishments, or characteristics. Inasmuch as action in social interaction is organized to minimize overt disagreement and misalignment and thus to promote mutual solidarity (e.g., Clayman, 2002), the family members’ actions could be seen to constitute a threat to it. From the perspective of the recognition theory, the situation looks quite different. In the mode of love/care, Jorma seems to receive special standing. The openness and directness of Liisa’s displays of disagreement, for example, can be seen as constructing their relationship as very close, since she is able to express herself in such a straightforward manner. Liisa’s laughter (l. 35, 37, 41) is not produced with a malicious tone but in a warm, teasing manner consistent with close family interactions. Liisa’s concluding assessment (“dad really is not always exactly on the same page,” l. 37–39), explicitly brings Jorma’s identity and membership category as the ‘out-of-touch dad’ of the family, as well as their long relationship history, to the surface of interaction. Jorma also embraces this identity and enacts it by mumbling “what” (l. 40). In our view, all this results in recognition of Jorma as a singular, irreplaceable individual with a special standing. This example thus brings to light how solidarity in the levels of action and recognition can be incongruent with each other, since here it is the dispreferred conversational actions that in effect are in service of recognition in the mode of love.

See Table 1 for a summary of our findings on how the different modes of recognition were implicated in the presented interactions.

**Table 1:** Organization of the analyzed data extracts in relation to the modes of recognition and their presence (present X; not present —).

Extract no.	Respect	Personalized esteem	Role-based/Category-mediated esteem	Love/care	Notes on (mis)recognition in the extract
Extract 1. Co-development workshop	—	—	—	—	Sequential deletion, non-ratification of participation
Extract 2. Decision-making in clubhouse	—	—	—	—	Minimal acknowledgement, denying status of “responsible agent”
Extract 3. Success story	X	X	—	—	Orientation to face-saving/personalized esteem
Extract 4. Cantor and pastor	X	—	X	—	Orientation to collegial relationship/role-based esteem
Extract 5. Shameful PhD supervision meeting	X	X	—	—	Orientation to interpersonal affiliation
Extract 6. Chalk paint	X	—	—	X	Orientation to irreplaceable individual

## 5 Discussion

Above we demonstrated how the three different modes of recognition can be at stake in face-to-face social interaction. Now we discuss our findings in relation to the specific research hypotheses presented at the beginning of this paper. In response to RQ1 (*How can we grasp recognition as an interactional phenomenon?*), we argue that recognition is actually implemented in and through social interaction. Our analysis considers recognition as a *momentary* phenomenon, which can vary from moment to moment. The small micro-moments of misrecognition can accumulate and create stronger and more severe processes of neglect or discrimination. At the same time, basic recognitive attitudes can be seen as more lasting dispositions of respect, love/care and esteem, which merely manifest themselves in situation-specific responses. For example loving someone can call for a response of joy when the loved one is doing well or being silly, and sadness and anguish when the loved one is suffering or in trouble. Behind the variety of situation-specific responses can be a lasting stance of recognizing the other. The basic relation between interaction and recognition is that interaction expresses, makes manifest as well as constitutes recognition. Interaction is the main way in which one can get experiences of being recognized.

In response to RQ2 (*How do the three modes of recognition show in interaction, either implicitly or explicitly?*), we have hopefully demonstrated through our examples how recognition is implicitly part of many different types of conversational activities and situations. In fact, we consider recognition to be part of *all* human interactions. Most often the business of recognition stays in the background, especially if due recognition is received, but sometimes it can rise to the surface-level of interaction. Even then, however, the demand for recognition is done in indirect ways, such as pursuing adequate recognition by repeating and recycling the same topical items (cf. Jefferson, 1978; see also ex. 3. and 4 in the current paper). The six examples we discussed show how respect (ex.1 and 2), esteem (ex. 3 and 4) and love/care (ex. 5 and 6) show up in interaction. It is important to note, however, that when we examine cases through a theoretical lens, such as recognition, and talk about potential but unrealized scenarios, there is a lot of room for differences in interpretation based on the analysts’ own life experiences and background. Still, we argue, recognition theory can sensitize the analyst to the different but relevant aspects that are not found in the participants’ talk. In this way, it is possible to refine the description of what the actual, realized scenario/conversational turn ultimately is doing.

In RQ3 we asked, how do conversational actions operate in relation to the recognition that they convey. As pointed out above, based on the CA theorizing on the social motives underlying the sequential organization of action, one would assume that the “solidarity-promoting” patterns of conversational actions would work in congruence with recognition, i.e., displays of affiliation and agreement would convey recognition and displays of disaffiliation and disagreement would convey misrecognition. However, as presented above, this is not the case. Firstly, even in moments of overt disagreement, the speaker is showing the co-participant respect—they are at least worthy of acknowledgement as communication partners. And as Extracts 5, 6 showed, sometimes displays of disagreement and actions that misalign with the co-participants project can be seen to convey a high level of recognition in the mode of love, even if not in the mode of esteem—or in the mode of esteem, even if not in the mode of love. And sometimes, the selection of criteria of esteem (when rival ones are available) can partly express solidarity or affiliation (ex.3).

The idea of distinguishing between the action level and recognition level of social interaction has an important implication for research. It is well known that conversation analysis shares the methodological commitment of social constructionism to “ontological muteness” (Nightingale and Cromby, 2002) regarding those aspects of social reality that go beyond the publicly observable features of interaction. The analysis should focus solely on how the participants *themselves* interpret each other’s behaviors as “morally accountable” (Garfinkel, 1967) actions, and the researcher is not supposed to produce any ontological claims detached from the participants’ own interpretations. However, the possibility of distinguishing recognition level from the level of action allows us to *theorize* also about those socially relevant interactional phenomena that go beyond the mechanisms of conditional relevance and the accountability of a next item upon the occurrence of a prior. This is important, as the sequential organization of interaction is intertwined with power relations that affect what different people can do in their interactions with others, how they can legitimately treat their interaction partners, and whether and when they can hold each other accountable for the deviations from the normative, expected or projected trajectory of interaction (Wetherell, 1998; Billig, 1999; Burr, 2015, p. 5). Indeed, we suggest that it is especially the violations at the recognition level of interaction that are particularly difficult—if not impossible—to raise to explicit reflective metalevel discussion, as this would necessitate the topicalization of social relations in a way that might become costly for the initiator of the discussion. In addition to this general difficulty, violations of recognition may be even more difficult to address by those individuals who have just previously been withheld recognition as fully legitimate participants and responsible agents in the encounter. Hence, to be able to also examine these critical phenomena we need to complement our empirical analysis with theorizing—and the recognition theory provides us with tools to do so.

If and when there is independent support for the central claims of recognition theory, and for seeing respect, esteem and love/care as central modes of recognition, it is possible to approach interaction sequences with the question of how is recognition manifested and constituted and renegotiated in the sequences of interaction. At the same time, a theorist of recognition can gain fresh insights from the cases. Example 3 concerned an achievement for an individual that typically might not count as much of an achievement. While both assessments are as such correct, the solidary thing to do is to choose the one that the achiever identifies with, or the one that allows the achiever to be seen in the good light, worthy of esteem. From the theorist’s armchair it might be difficult to anticipate such situations in which esteem and the selection of criteria of esteem make solidarity or lack of solidarity visible; even when solidarity is taken to cover both esteem and love/care. CA can thus bring the abstract ideas of recognition to life in concrete social situations and under detailed empirical analysis. Furthermore, CA can inform and promote the development of interactionally based social and societal critique by making visible some of the very subtle but significant moments of misrecognition involving, for example, ableism, sexism, or racism, and thus aid recognition theory in retaining its plausibility as a critical social theory (cf. Hirvonen and Koskinen, 2022). A more systematic study targeting ableism, sexism, or racism could try to detect recurrent patterns of interaction, with the hypothesis that such -isms lead to veridical experiences of misrecognition. In this article our aim has been to show that interaction and recognition are indeed deeply intertwined.

## Data availability statement

The original contributions presented in the study are included in the article, further inquiries can be directed to the corresponding author/s.

## Ethics statement

The studies involving humans were approved by different Finnish institutional ethics review boards. The studies were conducted in accordance with the local legislation and institutional requirements. The participants provided their written informed consent to participate in this study.

## Author contributions

EK, AL, and MS all contributed to conception of the study and wrote sections of the manuscript. All authors contributed to manuscript revision, read, and approved the submitted version.

## References

[ref1] Ásta (2018) Categories we live by. Construction of sex, gender, and other social categories. Oxford: Oxford University Press.

[ref2] Attwood T. (1998) Asperger's syndrome: a guide for parents and professionals. London: Jessica Kingsley

[ref3] Billig M. (1999). Whose terms? Whose ordinariness? Rhetoric and ideology in conversation analysis. Discourse Soc. 10, 543–558. doi: 10.1177/0957926599010004005

[ref4] Brown Penelope Levinson Stephen. (1987). Politeness. Some universals in language usage. Cambridge, UK: Cambridge University Press.

[ref5] Burr V. (2015). Social constructionism, 3rd ed. London: Routledge.

[ref6] Clayman S. E. (2002). “Sequence and solidarity” in Advances in group processes: group cohesion, trust, and solidarity. eds. Lawler E. J. Thye S. R. (Oxford, UK: Elsevier Science), 229–253.

[ref7] Dillon R. S. (2022). “Respect” in The Stanford encyclopedia of philosophy (fall 2022 edition). eds. Zalta E. N. Nodelman U.

[ref8] Durkheim E. (1933) in The division of labor in society. ed. Simpson G. (New York: Free Press)

[ref9] Garfinkel H. (1967). Studies in ethnomethodology. Englewood Cliffs, NJ: Prentice Hall.

[ref10] Goffman E. (1955). On face-work. Psychiatry 18, 213–231. doi: 10.1080/00332747.1955.11023008 13254953

[ref11] Goffman E. (1974). Frame analysis: an essay on the organization of experience. Boston: Northeastern University Press.

[ref9001] Haakana M. Laakso M. Lindström J. (eds.). (2009). “Introduction: comparative dimensions of talk in interaction,” in Talk in interaction: comparative dimensions. (Studia Fennica. Linguistica: Suomalaisen Kirjallisuuden Seura). 14, 15–47.

[ref12] Henry A. D. Barreira P. Banks S. Brown J. M. McKay C. (2001). A retrospective study of clubhouse-based transitional employment. Psychiatr. Rehabil. J. 24, 344–354. doi: 10.1037/h0095070, PMID: 11406985

[ref13] Hepburn A. Bolden G. (2013). “The conversation analytic approach to transcription” in The handbook of conversation analysis. eds. Sidnell J. Stivers T. (Chichester: Wiley)

[ref14] Heritage J. (1984). Garfinkel and ethnomethodology. Cambridge: Polity Press.

[ref15] Heritage J. (2008). “Conversation analysis as social theory” in The new Blackwell companion to social theory. ed. Turner B. S. (Blackwell: Oxford), 300–320.

[ref1001] Heritage J. (2011). “Territories of knowledge, territories of experience: empathic moments in interaction,” in The morality of knowledge in conversation. eds. T. Stivers, L. Mondada, and J. Steensig (Cambridge, UK: Cambridge University Press), 159–183.

[ref16] Hirvonen O. Koskinen H. J. (2022). The theory and practice of recognition. 1st Edn Routledge.

[ref17] Honneth A. (1995). The struggle for recognition. The moral grammar of social conflicts. Cambridge: Polity Press.

[ref18] Ikäheimo H. (2022). Recognition and the human life-form: beyond identity and difference Routledge.

[ref19] Jefferson G. (1978). “Sequential aspects of storytelling in conversation” in Studies in the Organization of Conversational Interaction. ed. Schenkein J. (New York: Academic Press, Inc), 219–248.

[ref20] Jefferson G. (1984). “On stepwise transition from talk about a trouble to inappropriately next-positioned matters” in Structures of social action. eds. Atkinson J. M. Heritage J. (Cambridge, UK: Cambridge University Press), 191–222.

[ref9002] Jefferson G. (2002). Is “no” an acknowledgment token? Comparing American and British uses of (+)/(–) tokens. J. Pragmat. 34, 1345–1383.

[ref21] Koskinen E. (2022). Storytelling, self, and affiliation: conversation analysis of interactions between neurotypical participants and participants with Asperger syndrome. Doctoral dissertation. University of Helsinki, Faculty of Social Sciences. Available at: https://helda.helsinki.fi/handle/10138/341931

[ref22] Koskinen E. Stevanovic M. Peräkylä A. (2021). The recognition and interactional Management of Face Threats: comparing Neurotypical participants and participants with Asperger's syndrome. Soc. Psychol. Q. 84, 132–154. doi: 10.1177/01902725211003023

[ref23] Laitinen A. (2002). Interpersonal recognition: a response to value or a precondition of personhood? Inquiry 45, 463–478. doi: 10.1080/002017402320947559

[ref9003] Laitinen A. (2015). “From recognition to solidarity: Universal respect, mutual support, and social unity,” in Solidarity: Theory and practice. eds. Laitinen A. Pessi A. B. (Lanham, MD: Lexington Books), 126–154.

[ref24] Linell P. (2009). Rethinking language, mind, and world dialogically. Interactional and contextual theories of human sense-making. Charlotte, NC: Information Age

[ref25] Maynard D. W. (2013). “Everyone and no one to turn to: intellectual roots and contexts for conversation analysis” in The handbook of conversation analysis. eds. Sidnell J. Stivers T., (Oxford, U.K: Wiley-Blackwell), 11–31.

[ref26] McBride C. (2014). Recognition. Cambridge: Polity Press.

[ref27] Murdoch Iris (1970). The sovereignty of good. London: Routledge and Kegan Paul.

[ref28] Nightingale D. J. Cromby J. (2002). Social constructionism as ontology: exposition and example. Theory Psychol. 12, 701–713. doi: 10.1177/0959354302012005901

[ref29] Pomerantz A. (1984). “Agreeing and disagreeing with assessments” in Structures of social action. eds. Atkinson J. M. Heritage J. (Cambridge, UK: Cambridge University Press), 57–101.

[ref31] Raeburn T. Halcomb E. Walter G. Cleary M. (2013). An overview of the clubhouse model of psychiatric rehabilitation. Australas. Psychiatry 21, 376–378. doi: 10.1177/1039856213492235, PMID: 23817899

[ref9004] Sacks H. (1992). Lectures on conversation. *Volumes I & II*. ed. Jefferson G.. Cambridge, UK: Blackwell.

[ref32] Sangiovanni A. Viehoff J. (2023). “Solidarity in social and political philosophy” in The Stanford encyclopedia of philosophy (summer 2023 edition). eds. Zalta E. N. Nodelman U..

[ref9005] Schegloff E. A. (2007). Sequence organization in Interaction: A primer in conversation analysis. *Volume 1*. Cambridge, UK: Cambridge University Press.

[ref9006] Sidnell J. Stivers T. (eds.) (2013). The handbook of conversation analysis. Malden, MA: Wiley-Blackwell.

[ref33] Siep L. Ikäheimo H. Quante M. (Eds.) (2022). Handbuch Anerkennung Springer. Reference Geisteswissenschaften. Springer VS, Wiesbaden.

[ref34] Sorjonen M.-L. (2001). Responding in conversation: a study of response particles in Finnish. Amsterdam/Philadelphia: John Benjamins.

[ref35] Stevanovic M. Peräkylä A. (2012). Deontic authority in interaction: the right to announce, propose and decide. Res. Lang. Soc. Interact. 45, 297–321. doi: 10.1080/08351813.2012.699260

[ref36] Stevanovic M. Peräkylä A. (2014). Three orders in the organization of human action: on the interface between knowledge, power, and emotion in interaction and social relations. Lang. Soc. 43, 185–207. doi: 10.1017/S0047404514000037

[ref37] Stevanovic M. (2012). Establishing joint decisions in a dyad. Discourse Stud. 14, 779–803. doi: 10.1177/1461445612456654

[ref38] Stivers T. (2008). Stance, alignment, and affiliation during storytelling: when nodding is a token of affiliation. Res. Lang. Soc. Interact. 41, 31–57. doi: 10.1080/08351810701691123

[ref39] Svennevig J. (2014). Direct and indirect self-presentation in first conversations. J. Lang. Soc. Psychol. 33, 302–327. doi: 10.1177/0261927X13512307

[ref40] Taylor C. (1992). Ethics of authenticity. Cambridge, MA: Harvard University Press.

[ref41] Taylor C. (1995). ‘Politics of recognition’, in philosophical arguments. Cambridge, MA: Harvard University Press.

[ref42] Thompson S. (2006). The political theory of recognition: a critical introduction. Cambridge: Polity Press.

[ref43] Valkeapää T. Tanaka K. Lindholm C. Weiste E. Stevanovic M. (2019). Interaction, ideology, and practice in mental health rehabilitation. J. Psychosoci. Rehab. Mental Health 6, 9–23. doi: 10.1007/s40737-018-0131-3

[ref44] Weiste E. Stevanovic M. Uusitalo L.-L. I. (2022). Experiential expertise in the co-development of social and health-care services: self-promotion and self-dismissal as interactional strategies. Sociol. Health Illn. 44, 764–780. doi: 10.1111/1467-9566.13457, PMID: 35352357 PMC9311060

[ref45] Wetherell M. (1998). Positioning and interpretative repertoires: conversation analysis and post-structuralism in dialogue. Discourse Soc. 9, 387–412. doi: 10.1177/0957926598009003005

