# Supplementary material for: Recognition in interaction: theoretical and empirical observations
Source: Front Sociol. 2024 Jan 23;8:1223203. doi: 10.3389/fsoc.2023.1223203 (PMC10844475; doi:10.3389/fsoc.2023.1223203)
Supplement: Supplementary file 1 [file Data_Sheet_1.docx]

**Appendix A. Transcription Symbols**

[ ] overlapping talk

= latching

(.) micro pause

(0.1) timed pause

- cut-off of preceding sound

: extension of a sound

°word° quieter voice

WORD louder voice

>word< faster talk

<word> slower talk

€word€ smiley voice

#word# creaky voice

.hh aspiration

hh out breath

word emphasis

w(h)ord(h) laugh particles

↑↓ rise or fall in pitch

? rising intonation

, continuing intonation

. falling intonation

(---) transcriber could not hear what was said

((sitting)) transcriber’s descriptions of phenomena

{___} beginning, end and duration of the described phenomenon

identified participant’s multimodal actions

**Appendix B. Glossing Abbreviations**

PL plural

1, 2 person

DEM demonstrative pronoun

POSS possessive suffix

GEN genitive

PAR partitive

ESS essive

TRA translative

INE inessive

ELA elative

ILL illative

ADE adessive

ABL ablative

ALL allative

COMP comparative

PRT particle

CLI clitic

Q question (clitic) particle

INF infinitive

COND conditional

IMP imperative

PASS passive

PST past tense

PPC past participle

PPPC passive past participle
